# Beyond LDL-C: unravelling the residual atherosclerotic cardiovascular disease risk landscape—focus on hypertriglyceridaemia

**DOI:** 10.3389/fcvm.2024.1389106

**Published:** 2024-08-07

**Authors:** Bilal Bashir, Jonathan Schofield, Paul Downie, Michael France, Darren M. Ashcroft, Alison K. Wright, Stefano Romeo, Ioanna Gouni-Berthold, Akhlaq Maan, Paul N. Durrington, Handrean Soran

**Affiliations:** ^1^ Faculty of Biology Medicine and Health, University of Manchester, Manchester, United Kingdom; ^2^ Department of Endocrinology, Diabetes & Metabolism, Manchester University NHS Foundation Trust, Manchester, United Kingdom; ^3^ NIHR/Wellcome Trust Clinical Research Facility, Manchester, United Kingdom; ^4^ Department of Clinical Biochemistry, Bristol Royal Infirmary, Bristol, United Kingdom; ^5^ Department of Clinical Biochemistry, Central Manchester University Hospitals, NHS Foundation Trust, Manchester, United Kingdom; ^6^ Centre for Pharmacoepidemiology and Drug Safety, Division of Pharmacy and Optometry, School of Health Sciences, Faculty of Biology, Medicine and Health, University of Manchester, Manchester, United Kingdom; ^7^ Department of Molecular and Clinical Medicine, University of Gothenburg, Gothenburg, Sweden; ^8^ Clinical Nutrition Unit, Department of Medical and Surgical Sciences, Magna Graecia University, Catanzaro, Italy; ^9^ Cardiology Department, Sahlgrenska University Hospital, Gothenburg, Sweden; ^10^ Centre for Endocrinology, Diabetes and Preventive Medicine, Faculty of Medicine and University Hospital Cologne, University of Cologne, Cologne, Germany

**Keywords:** hypertriglyceridaemia, cardiovascular risk, fibrates, omega-3 fatty acids, statins, atherosclerosis, residual risk

## Abstract

**Aims:**

Historically, atherosclerotic cardiovascular disease (ASCVD) risk profile mitigation has had a predominant focus on low density lipoprotein cholesterol (LDL-C). In this narrative review we explore the residual ASCVD risk profile beyond LDL-C with a focus on hypertriglyceridaemia, recent clinical trials of therapeutics targeting hypertriglyceridaemia and novel modalities addressing other residual ASCVD risk factors.

**Findings:**

Hypertriglyceridaemia remains a significant ASCVD risk despite low LDL-C in statin or proprotein convertase subtilisin/kexin type 9 inhibitor-treated patients. Large population-based observational studies have consistently demonstrated an association between hypertriglyceridaemia with ASCVD. This relationship is complicated by the co-existence of low high-density lipoprotein cholesterol. Despite significantly improving atherogenic dyslipidaemia, the most recent clinical trial outcome has cast doubt on the utility of pharmacologically lowering triglyceride concentrations using fibrates. On the other hand, purified eicosapentaenoic acid (EPA), but not in combination with docosahexaenoic acid (DHA), has produced favourable ASCVD outcomes. The outcome of these trials suggests alternate pathways involved in ASCVD risk modulation. Several other pharmacotherapies have been proposed to address other ASCVD risk factors targeting inflammation, thrombotic and metabolic factors.

**Implications:**

Hypertriglyceridaemia poses a significant residual ASCVD risk in patients already on LDL-C lowering therapy. Results from pharmacologically lowering triglyceride are conflicting. The role of fibrates and combination of EPA and DHA is under question but there is now convincing evidence of ASCVD risk reduction with pure EPA in a subgroup of patients with hypertriglyceridaemia. Clinical guidelines should be updated in line with recent clinical trials evidence. Novel agents targeting non-conventional ASCVD risks need further evaluation.

## 1 Introduction

Atherosclerotic Cardiovascular Disease (ASCVD) remains the leading cause of morbidity and mortality worldwide despite new mechanistic insights and preventative strategies to mitigate ASCVD risk. Increased prevalence of conditions that predispose to ASCVD events i.e., obesity, diabetes, hypertension and atherogenic dyslipidaemia contributes to the increasing burden of care attributed to ASCVD that costs >200 billion US dollars annually for US and comparable figure for Europe (1, 2). Low-density lipoprotein cholesterol (LDL-C) has been demonstrated in large genetic, epidemiological, and clinical studies as a leading cause of atherosclerosis and ASCVD (3, 4). A meta-regression analysis of 26 randomised controlled trials (RCTs) has demonstrated a stepwise reduction in ASCVD risk with 22% relative risk reduction with each 1 mmol/L reduction in LDL-C (5). Patients with higher pre-treatment LDL-C benefit more (6) and there is no limit below which further LDL-C lowering ceases to confer ASCVD protection (7). Despite this, there remains significant residual risk in statin treated patients (8–13). Addition of pharmacotherapies like proprotein convertase subtilisin/kexin type 9 (PCSK9) inhibition can reduce LDL-C to a very low level. Despite achieving LDL-C of <30 mg/dl (0.7 mmol/L) a substantial number of individuals still experience ASCVD events (14). In this narrative review, we delve into residual cardiovascular risks that extend beyond LDL-C with a focus on the role of triglyceride rich lipoproteins (TRL) as a residual ASCVD risk factor and on the impact of triglyceride (TG) lowering pharmacotherapy on residual ASCVD risk.

## 2 Methods

We conducted a comprehensive search across multiple electronic databases, including AMED, Embase, HMIC, Pubmed, Ovid Emcare, Ovid MEDLINE and other relevant papers of interest collected by the authors. Our search strategy utilized the following terms: “HYPERTRIGLYCERIDAEMIA”, “FIBRATES”, “RESIDUAL RISK”, “RANDOMISED CONTROLLED TRIALS”, “OMEGA 3 FATTY ACID”, “CARDIOVASCULAR”, “TRIGLYCERDE RICH LIPOPROTEINS” and “TRIGLYCERIDE”. Boolean operators “AND” and “OR” were employed to combine and separate search terms effectively. Only articles published in the English language were considered for inclusion in this review. Exclusion criteria encompassed articles published in languages other than English, conference abstracts, and case reports. Additionally, only studies involving human participants were included. To supplement our database search, we manually scrutinized the reference lists of identified trials, review articles, and previous meta-analyses to identify any additional relevant data.

## 3 Residual atherosclerotic cardiovascular disease risk

### 3.1 Residual ASCVD risk in clinical trials

The Further cardiovascular OUtcomes Research with PCSK9 Inhibition subjects with Elevated Risk (FOURIER) trial evaluated patients with known ASCVD with pre-treatment LDL-C of 2.4 mmol/L (92 mg/dl) that was reduced to 0.7 mmol/L (30 mg/dl) with evolocumab, 9.8% of drug recipients still experienced ASCVD events over median followup period of 2.2 years (14). Similarly, in the Evaluation of Cardiovascular outcomes after Acute coronary syndrome during treatment with Alirocumab (ODYSSEY Outcomes), despite achieving LDL-C as low as 1.7 mmol/L (66 mg/dl), probability estimate of alirocumab recipients to have ASCVD event was 12.8% at 4 years (15). There were similar results in the Studies of PCSK9 Inhibition and the Reduction of Vascular Events (SPIRE) trials, though the relative percentage of ASCVD events was lower than FOURIER and ODYSSEY Outcomes (2.1% in SPIRE 1 at median followup of 7 months and 3.4% in SPIRE 2 at median followup of 12 months) (16) In the landmark statin trials where LDL-C lowering reduced the relative risk of ASCVD, participants who received statins still exhibited a significant residual cardiovascular risk. This was 22.4% at 2 years in the Pravastatin or Atorvastatin Evaluation and Infection Therapy–Thrombolysis in Myocardial Infarction 22 (PROVE IT-TIMI) study (achieved LDL-C 62 mg/dl, 1.6 mmol/L) (17), 9.3% over median follow up of 4.8 years in the Incremental Decrease in End Points Through Aggressive Lipid Lowering (IDEAL) study (achieved LDL-C 81 mg/dl, 2.1 mmol/L) (18), and 8.7% over a median follow up of 4.9 years in the Treating to New Targets (TNT) study (achieved LDL-C 77 mg/dl, 1.9 mmol/L) (19). That a heightened incidence of ASCVD events persists despite attainment of low levels of LDL-C prompted the conceptualisation of residual cardiovascular risk due to additional metabolic, inflammatory, and thrombotic risk factors. The mitigation of ASCVD events necessitates a comprehensive and multifaceted approach addressing these diverse components.

### 3.2 Lipoproteins in atherosclerosis—limitations of LDL-C calculation

The key initial event in the genesis of atherosclerosis is the entrapment of lipoproteins in vascular intima followed by the engulfment by macrophages. Lipoprotein subfractions other than LDL can be preferentially entrapped and engulfed without the need to be chemically modified and may enhance the process of atherosclerosis. While the conventional clinical approach employs LDL-C as a marker for atherosclerotic risk, many atherogenic particles are relatively deficient in cholesterol and so their atherogenicity is underestimated by cholesterol measurement. In most clinical laboratories, LDL-C is an estimated through the Friedwald formula, dependant on knowledge of the total cholesterol (TC), High density lipoprotein cholesterol (HDL-C) and TG concentration. With TG >4.5 mmol/L (400 mg/dl) an estimate for LDL-C cannot be provided by this formula and even modest excursions in TG concentration, will result in underestimation of LDL-C concentration. Alternative calculators are available that offer LDL-C estimations up to TG levels of 10 mmol/L (20).

### 3.3 Apolipoprotein B100 and lipoprotein sub-fractions as a marker for ASCVD risk estimation

Apolipoprotein B100 (ApoB) serves as a comprehensive metric for the total atherogenic particle count. Each atherogenic lipoprotein particle, such as LDL, very low-density lipoprotein (VLDL), and Intermediate Density Lipoprotein (IDL), contains a single ApoB molecule. Consequently, the blood ApoB level provides a direct reflection of the overall number of atherogenic particles, irrespective of their size or density. This establishes ApoB as a more precise indicator of ASCVD risk compared to traditional lipid measurements, which fail to consider particle number or size. The Apolipoprotein-related Mortality Risk (AMORIS) study found that ApoB levels and the ApoB/Apolipoprotein A1 ratio were stronger predictors of ASCVD than LDL-C, whilst TG was found to be an independent risk factor for ASCVD (21). In the *post hoc* analysis of the TNT trial, higher levels of TRL (VLDL, IDL and chylomicron remnants) were associated with an increased risk of major ASCVD events independent of LDL-C concentration (22). Mendelian randomisation studies, epidemiological observations and RCTs of lipid-lowering drugs have implicated cholesterol-rich ApoB particles in addition to LDL i.e., VLDL, IDL and Lipoprotein (a) [Lp(a)] as being directly causal in ASCVD (4). In a prospective observational study of 4,932 individuals from the Jackson Heart Study and the Framingham Offspring Cohort Study, free of coronary heart disease (CHD) at baseline followed-up for 8 years, remnant lipoprotein cholesterol (RLP-C) was linked to the onset of CHD. After adjusting for other ASCVD risk factors and HDL-C, this association was driven by IDL-C which significantly elevated CHD risk by 25% (HR 1.25, 95% CI 1.07–1.46, *P* < 0.001) (23).

Variations in the *Lipoprotein lipase (LPL)* gene that augment LPL activity are correlated with reduced TG levels and a concomitant decrease in ApoB concentration. Variations in the *low-density lipoprotein receptor (LDLR)* gene that enhance the activity of the LDLR are linked to decreased LDL-C concentration and a corresponding reduction in ApoB. For every 10 mg/dl decline in plasma ApoB concentration attributable to LPL score-associated variants, a parallel decrease of 0.8 mmol/L (69.9 mg/dl) in TG concentration is observed, with no discernible alteration in LDL-C, and a diminished risk of CHD (odds ratio (OR), 0.771 [95% CI, 0.741–0.802]). An equivalent 10 mg/dl decrease in plasma ApoB concentration associated with LDLR score-related variants corresponds to a 0.4 mmol/L (14.1 mg/dl) reduction in LDL-C concentration, no alteration in TG, and a similarly decreased risk of CHD [OR 0.773 (95% CI, 0.747–0.801)]. Consequently, despite inducing modifications in distinct lipid profiles, both LPL and LDLR scores exhibit analogous reductions in CHD risk for the same decrement in plasma ApoB concentration (24). This underscores that in hytriglyceridaemic populations ApoB is a better predictor of cardiovascular risk than cholesterol-based parameters, and a pivotal treatment target (25). There has been accumulating evidence recently which suggest that the risk attributed to an incremental rise in TRL/remnant cholesterol surpasses that of an equivalent increase in LDL-C (26). This was elaborated more recently in a Bjornsen et al. in a well characterised population from the UK Biobank. The authors investigated 502,460 participants in the UK Biobank, examining all single nucleotide polymorphisms (SNPs) associated with TRL and LDL-C identified via genome wide association studies and standard lipid profiles, including ApoB. These SNPs were divided into 2 clusters. Cluster 1 included SNPs affecting receptor mediated clearance and hence LDL-C more than TRL/remnant cholesterol, while cluster 2 had SNPs with a stronger impact on lipolysis and hence TRL/remnant cholesterol. The OR for CHD per standard deviation (SD) increase in ApoB was 1.76 (95% CI: 1.58–1.96) in cluster 2, which was significantly higher than the OR in cluster 1 [1.33 (95% CI: 1.26–1.40)]. These findings suggest that the association of ApoB with CHD risk varies depending on the type of particle harbouring ApoB and in this study, TRL/remnant particles demonstrated significantly greater atherogenicity per particle compared to LDL (27).

Despite low ApoB concentration, rare cases of familial dysbetalipoproteinaemia (FDBL) with ApoE2 homozygosity exhibited heightened ASCVD risk (28). This is attributed to impaired liver processing of chylomicron remnants, leading to prolonged circulation and abnormal cholesterol enrichment due to cholesteryl ester transfer protein (CETP) mediated lipid exchanges. Generating atherogenic small-dense LDL particles, which does not figure well in LDL-C measurements, in patients with high TG level as well as TG's strong association with atherogenic components of the metabolic syndrome, high-sensitivity C-reactive protein (hsCRP), coagulation are other factors contribute to increased ASCVD risk in hypertriglyceridaemia (29–34). It is now accepted that using ApoB to assess ASCVD risk in hypertriglyceridaemia (2–10 mmol/L) better reflects the total number of atherogenic particles than do LDL-C or non-HDL-C, particularly in patients with hypertriglyceridemia (25, 29–36).

### 3.4 Severe hypertriglyceridaemia and ASCVD

There is an apparent paradox that hypertriglyceridaemia >10 mmol/L (885 mg/dl) association with ASCVD risk is less than less severe hypertriglyceridaemia. In the CALIBER study, Patel and colleagues, found no increased risk of myocardial infarction (MI) in individuals with TG >10 mmol/L (885 mg/dl), while increased risk was found in mild to moderate hypertriglyceridaemia (1.7–10.0 mmol/L, 150–885 mg/dl) that persisted despite statin and/or fibrate treatment (37). This is consistent with the notion that small but numerous TG depleted particles are more atherogenic that large TG rich particles such as chylomicrons. TG >10 mmol/L (885 mg/dl) is often associated with chylomicronaemia with particles enriched in TG relative to ApoB. Monogenic disorders causing severe hypertriglyceridemia have increased chylomicron concentrations with a heightened risk of acute pancreatitis, but generally not of premature atherosclerosis, likely due to the limited ability of chylomicrons to traverse the vascular endothelial barrier (38). Additionally, in severe hypertriglyceridemia (TG >10.0 mmol/L; 885 mg/dl) ApoB immunoassays are compromised by analytical interference in blood samples due to turbidity caused by large chylomicron and VLDL particles (39). TG measurement does not, therefore, reflect an increased number of atherogenic particles in these cases. Indeed, chylomicron associated ApoB generally contributes very little to total plasma ApoB.

**Figure 1:**
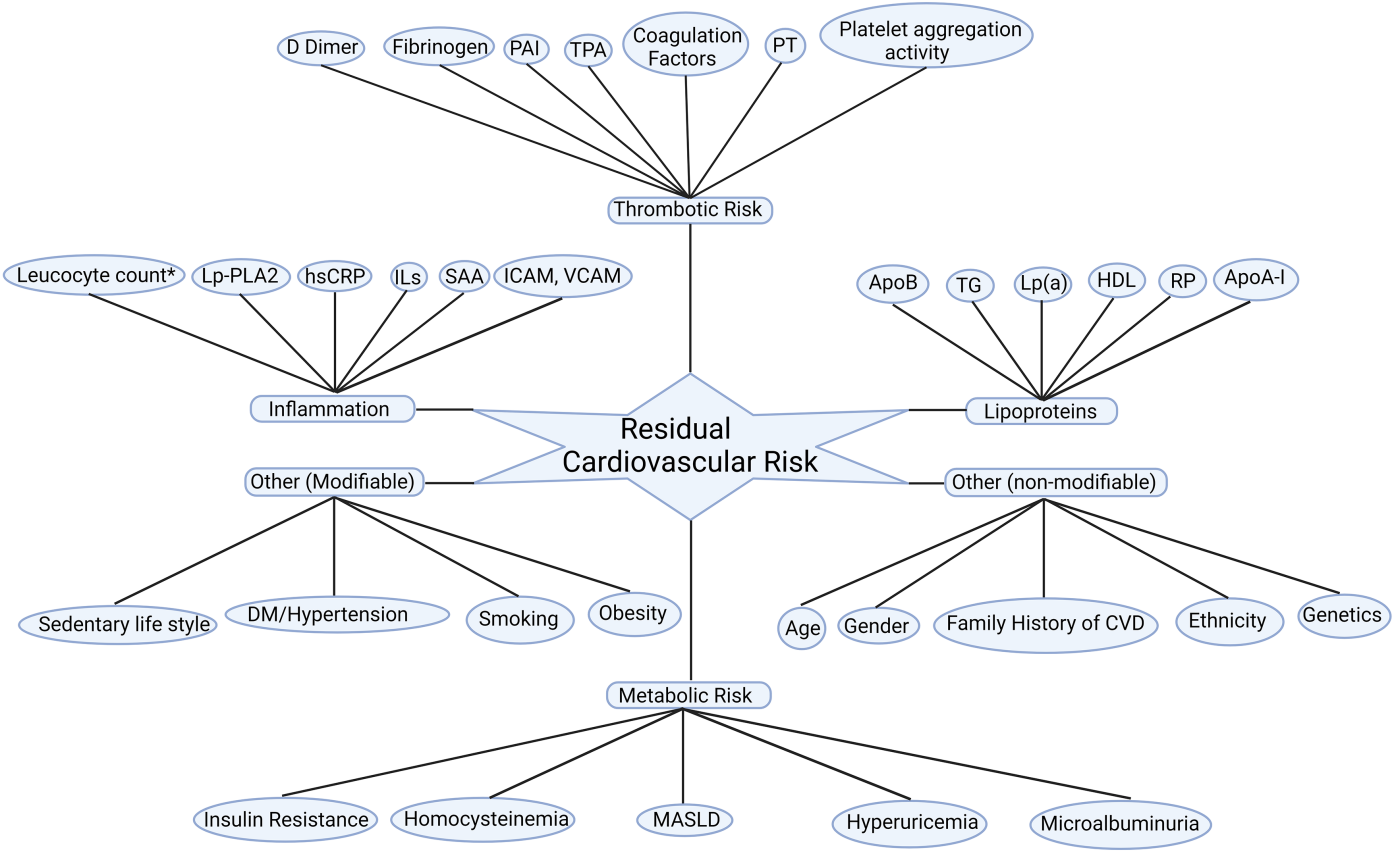
Residual cardiovascular risk factors. Apo A-I, apolipoprotein A-I; ApoB, apolipoprotein B100; CVD: cardiovascular disease; DM, diabetes mellitus; HDL-C; high-density lipoprotein cholesterol; hsCRP, high sensitivity C reactive protein; ICAM, intercellular adhesion molecule; ILs, interleukins; Lp(a), lipoprotein (a); Lp-PLA_2_, lipoprotein-associated phospholipase A_2_; MASLD: metabolic dysfunction-associated steatotic liver disease; PAI, plasminogen activator inhibitor; PT, prothrombin time; RP, remanent particles; SAA, serum amyloid A; TG, triglycerides; TPA, tissue plasminogen activator; VCAM; vascular cell adhesion molecule. *Leucocyte count after acute myocardial infarction.

In addition to various lipid subfractions, other metabolic, inflammatory contribute to and thrombotic pathways also fuel residual ASCVD risk as summarised in Figure 1.

## 4 Triglycerides as a residual ASCVD risk factor

### 4.1 Definition and measurement of triglycerides

Despite heterogeneity in the definition of hypertriglyceridaemia, normal levels of fasting TG have been defined at <1.7 mmol/L (<150 mg/dl) (40–42). Persistent hypertriglyceridaemia is defined as a fasting TG ≥1.7 mmol/L (≥150 mg/dl) following a minimum of 4–12 weeks of lifestyle intervention, a stable dose of maximally tolerated statin when indicated as well as evaluation and management of secondary causes of hypertriglyceridaemia (41). More recently a more stringent criteria of TG <1.2 mmol/L (100 mg/dl) has been proposed to define optimal TG concentration (43). There has been discrepancy in recommendations between different guidelines regarding fasting or non-fasting lipid measurements for ASCVD risk assessment (43–46). Non-fasting and fasting samples provide comparable results for TC, LDL-C and HDL-C. The concentration of TG is elevated during the postprandial phase (40) though the increment is modest in majority of patients, between 0.14–0.3 mmol/L (12–27 mg/dl) (41) Non-fasting rather than fasting TG concentration is independently associated with atherosclerosis and incident future ASCVD events independent of other ASCVD risk factors, lipid parameters and insulin resistance (45, 47, 48). Using non-fasting samples of 6,391 participants in the Women's Health Study, a cut-off of 1.98 mmol/L (175 mg/dl) has been proposed to predict future ASCVD events (49). Fasting and non-fasting TG was found to be in good agreement in the Anglo-Scandinavian Cardiac Outcome Trial (ASCOT-LLT) with no difference in ASCVD outcomes between both groups (50). Most of the lipid modification clinical trials in last couple of decades used fasting lipid samples, though the implication of postprandial lipaemia and delayed clearance of TRL in postprandial state on ASCVD risk was conceptualised as early as 1979 (51) and has been subsequently tested in several clinical studies where postprandial TG was better predictor of ASCVD risk (52–54). This could be due to remnant particles that contribute to atherogenesis and are better captured in non-fasting samples. Humans are in a postprandial state most of the time during the day and therefore, a postprandial lipid profile may prove to be a more reliable and physiological marker of future ASCVD risk.

### 4.2 Hypertriglyceridaemia and ASCVD—mechanism and implications

**Figure 2:**
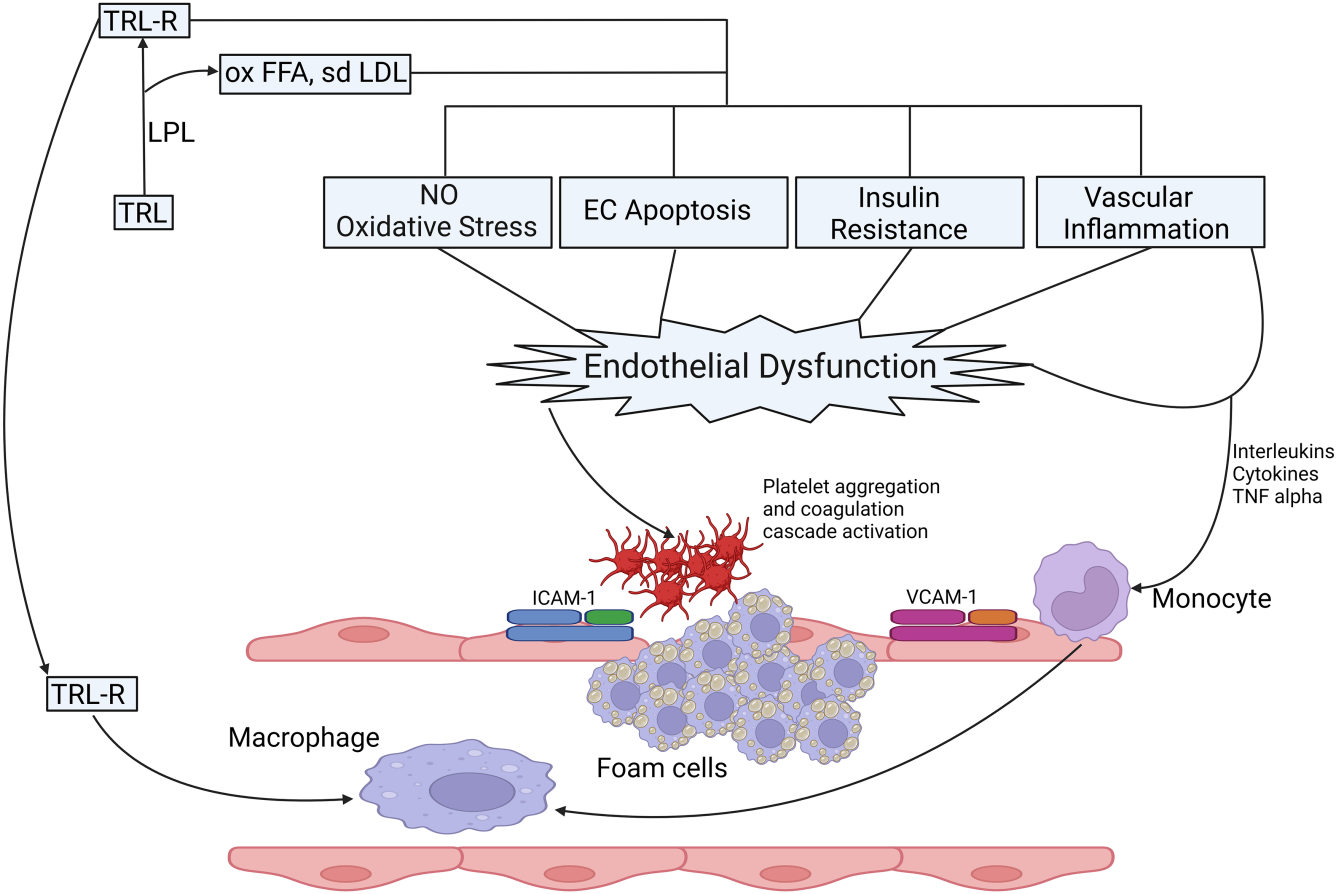
Possible mechanisms of TRLs in the process of the onset and progression of atherosclerosis. Catabolism of TRL leads to the production of FFA, sdLDL and their oxidized products, oxidized FFA and ox-sdLDL and remnant particles. Catabolic products of triglycerides increase the production of ROS, increase oxidative stress, reduce NO production, induce EC apoptosis, effects insulin signalling leading to IR, vascular inflammation the expression of proinflammatory cytokines and upregulate endothelial expression adhesion molecules (ICAM-1 and VCAM-1) facilitating the migration of proinflammatory leucocytes to enhance inflammatory response (55). Retention of TRL lipoproteins, remanent particles and breakdown products in vascular intima attract monocytes that differentiate into macrophages leading to the production of foam cells after engulfing TRL and remanent particles, which form the core of atherosclerotic plaque. The proinflammatory milieu leads to aggregation and activation of platelets and coagulation cascade, thereby inducing a pro-coagulant state and clot formation (55, 56, 60). CAM, intercellular adhesion molecule; EC, endothelial cells; FFA, free fatty acid; IR, insulin resistance; ICAM, inter-cellular adhesion molecule; LPL, lipoprotein lipase; NO: nitric oxide; ox, oxidised; sdLDL, small dense LDL; TNF, Tumour necrosis factor; TRL, triglyceride rich lipoproteins; TRLR, triglyceride rich lipoprotein remnants; VCAM, vascular cell adhesion molecule.

With the rising prevalence of obesity, diabetes, insulin resistance and metabolic syndrome, evidence to suggest a causal relationship between hypertriglyceridemia and ASCVD has been accumulating. Catabolism of TRL leads to the liberation of remnant particles, small dense LDL particles (sdLDL), HDL3 and free fatty acids (FFA) (55, 56). FFA have a multidimensional role that triggers endothelial dysfunction through oxidative stress, impaired nitric oxide (NO) production, inflammation, and endothelial cell apoptosis (57–59) (Figure 2). Remnant particles, which are lipolytic products of chylomicrons and VLDL, vary in size and composition. They are smaller than their parent molecule and have a greater cholesterol-to-TG ratio. Increased production of VLDL and slower clearance of remnant particles and VLDL due to reduced LPL activity delays their conversion to downstream lipoprotein particles (38) thereby increasing their circulatory time. Similarly, inability of hepatic receptors to clear them from the circulation e.g., in individuals harbouring homozygous *Apolipoprotein E2* isoform (FDBL) increases the time spent in the systemic circulation. With increased circulatory time, they are more likely to be entrapped in vascular intima, and in contrast to LDL, can be taken up by macrophages without chemical modifications, facilitating the process of atherogenesis (61). Apolipoprotein B48, a lipoprotein-associated with gut-derived chylomicron particles and chylomicron remnants has been found in atherosclerotic plaques derived from human aortic, carotid, and femoral endarterectomy specimens (62, 63).

### 4.3 Complex association between hypertriglyceridemia and atherosclerotic cardiovascular disease: the confounding role of HDL-C

Despite large-scale epidemiological and population-based studies suggesting the association of hypertriglyceridaemia with ASCVD (Table 1), unlike LDL-C, it has been a challenge to establish the causal role of TG with ASCVD. The difficulty in establishing this causal link partly stems from the inverse relationship between TG and HDL-C (73) and progressively increasing levels of RLP-C and density of LDL with increasing TG: HDL-C ratio (74) thereby confounding the independent effect of hypertriglyceridaemia on ASCVD. Recent epidemiological studies have attempted to address this by studying the effect of hypertriglyceridaemia in patients with LDL-C <2.6 mmol/L (100 mg/dl), a routinely accepted target in people who are at moderate risk of future ASCVD event (75). In a population-based study of 27,953 statin-treated patients from the Pacific Northwest and Southern California, with LDL-C 1.0–2.6 mmol/L (40–100 mg/dl), hypertriglyceridaemia (200–499 mg/dl, 2.2–5.6 mmol/L) was found to independently increase the risk of nonfatal MI and coronary revascularisation over an average follow-up period of 5.3 years (70). Similar findings were observed in a primary prevention cohort with diabetes (*n* = 28,318) and LDL-C <2.6 mmol/L (100 mg/dl) where the risk of CHD was found to be higher in the cohort with hypertriglyceridaemia (TG >150 mg/dl, 1.7 mmol/L) and low HDL-C (≤50 mg/dl, 1.3 mmol/L and ≤40 mg/dl, 1.0 mmol/L for women and men respectively), [Women: HR 1.35 (1.14–1.60), Men: HR 1.62 (1.43–1.83)]. This difference was significant only in women in the cohort with low HDL-C and high TG and highest in men in the same group when compared to low HDL-C, normal TG or normal HDL-C, high TG (76). Similarly, hypertriglyceridaemia (>150 mg/dl, 1.7 mmol/L) has been demonstrated to be associated with subclinical atherosclerosis regardless of baseline LDL-C levels, [LDL-C <100 mg/dl, 2.6 mmol/L OR: 1.85 (1.08–3.18), LDL-C >100 mg/dl, 2.6 mmol/L OR: 1.42 (1.11–1.80)] and vascular inflammation (12).

**Table 1:** Major studies evaluating the effect of hypertriglyceridaemia on ASCVD outcomes.

Study	Type of study	Study population	Location	No of participants	Fasting or non-fasting	Follow up (years)	Outcomes	Comments
Copenhagen City Heart Study (64, 65)	Prospective population-based study	Primary prevention	Denmark	13,981	Non-fasting	26	Incident MI IHD Total death Ischemic stroke	Increased risk of MI, IHD, Ischemic stroke and total death with hypertriglyceridaemia group. Each 1 mmol/L rise in TG is associated with a 10%–24% increased risk of MI, IHD, ischemic stroke and death.
Bansal et al. (45)	Prospective study of cohort derived from Women's Health Study^a^	Primary prevention	USA	26,509	Fasting: 20,118 Non-fasting: 6,391	11.4	MI/ischemic stroke/coronary revascularization/or ASCVD death.	Non-fasting TG levels (2–4 h after a meal) were strongly associated and were a better predictor of future cardiovascular events, independent of other ASCVD risks and HDL-C level, [HR 1.98, (1.21–3.25), *p* = 0.006]
Hokanson et al. (66)	Meta-analysis of 17 population-based, prospective studies (European and American)	NR	Europe & America	57,277	Fasting	9.9	MI CHD IHD ASCVD Death	1 mmol/L increase in TG is associated with 32% increased risk of ASCVD event in men [RR 1.32 (1.26–1.39)] and 76% in women [RR 1.76, (1.50–2.07)]. Adjustment for HDL-C and other risk factors attenuated the risk to 14% in men (RR 1.14, CI 1.05–1.28) and 37% in women [RR 1.37, (1.13–1.66)]
Patel et al. (67)	Meta-analysis of 26 prospective cohort studies (Asia and Pacific region)	NR	Asia, Australia, New Zealand.	96,224	Fasting in 90% population	8.2	CHD Stroke	Compared to TG ≤0.7 mmol/L, 1.1–1.3 mmol/L was associated with a 30%–50% higher risk of ASCVD event. The highest TG tertile (≥1.9 mmol/L), after adjusting for other risk factors, was associated with a 70%–80% higher risk of CHD event. Each 1-SD-higher level of log-triglycerides led to a greater risk of fatal CHD [HR 1.33, (1.09–1.62)] and fatal or nonfatal CHD [HR 1.56, (1.20–2.03)].
Reykjavik study (68)	Prospective population-based cohort study	Primary prevention	Iceland	18,569	Fasting	17.4	Non-Fatal MI CHD Death	Each log unit increase in TG level (mmol/L) is associated with a 40% increase in the risk of fatal or non-fatal MI for women [HR 1.40, (1.15–1.70)] and 21% in men [HR 1.21, (CI 1.08–1.36)]. The multivariate model did not adjust for HDL-C.
Sarwar et al. (69)	A case-control study from cohorts derived from the Reykjavik study and EPIC-Norfolk Study	Primary prevention	Iceland and England	Reykjavik: Case: 2,459 Controls: 3,969	Fasting	20	CHD	People with TG in the top tertile (>1.28 mmol/L) are more likely to experience CHD events as compared to individuals in the bottom tertile (<0.87 mmol/L), OR 1.43 (1.23–1.65), though attenuated but stayed significant after adjusting for other CV risk factors. Adjusted values for HDL-C are not available.
				Epic-Norfolk: Cases: 1,123 Controls: 2,206	Non-fasting	8	CHD	People with TG in the top tertile (>2.0 mmol/L) are more likely to experience CHD events as compared to individuals in the bottom tertile (<1.33 mmol/L), OR 1.52 (1.24–1.89). Adjusted OR for HDL-C in addition to traditional CV risk factors, 1.31 (1.06–1.62)
Sarwar et al. (69)	Meta-analysis of 29 population-based, prospective studies, including Reykjavik and EPIC-Norfolk Studies (European and American)	NR	Europe & America	262,525	Fasting: 23 studies (*n* = 119,044) Non-Fasting: 6 studies (*n* = 143,481)	12.1	CHD	OR for CHD in individuals with usual TG values in the top third of the population compared with those in the bottom third, adjusted for several established risk factors, was 1.7 (1.6–1.9). Adjustment for HDL-C attenuated but did not eliminate the risk of CHD with high TG.
Nichols et al. (70)	Population-based cohort study	Secondary prevention or >50 years with diabetes and one more ASCVD risk factor	USA	27,953	Both fasting and non-fasting	5.3	Non-fatal MI Non-fatal Stroke UA Coronary revascularisation	Compared to normal TG (<1.7 mmol/L) hypertriglyceridemia (2.2–5.6 mmol/L) at optimum LDL-C (1.0–2.6 mmol/L), the incidence rate of non-fatal MI, non-fatal stroke and coronary revascularisation was 30%, 23% and 21% higher in hypertriglyceridemia group both [RR 1.30, (1.08–1.58), 1.23 (1.01–1.49) and 1.21 (1.02–1.43) respectively]. No difference between groups for UA [RR 1.33, (0.87–2.03)].
TG REAL (71)	Population-based cohort study	Primary prevention	Italy	15,8072	Predominantly fasting	3.2	Incident ASCVD event All-cause mortality	As compared to normal TG (<1.7 mmol/L), high TG (1.7–5.6) and very high TG (>5.6), after adjusting for confounders, were associated with a higher risk of incident ASCVD [HR 1.61, (1.43–1.82) and 2.30 (1.02–5.18) respectively] and all-cause mortality [HR 1.49, (1.36–1.63) and HR 3.08, (1.46–6.50) respectively].
Lee et al. (72)	Population-based cohort study	Primary prevention	South Korea	5,688,055	NR	7.1	MI Stroke All-cause death	The predictive value of TG was strongest amongst all lipid components that did not attenuate after adjusting for conventional CV risk factors. Triglycerides in the highest quantile (≥1.7 mmol/L) were independently associated with the risk of ASCVD as composite [HR 2.08, (2.02–2.15)] or components of composite, [MI: HR 2.48, (2.33–2.64) or Stroke: HR 2.53 (2.34–2.73)].
Patel et al. (37)	Population-based cohort study	Primary and secondary prevention	United Kingdom	1,530,441	NR	6.7	AMI All-cause mortality	As compared to normal triglycerides (<1.7 mmol/L) individuals with mild (1.7–4.5 mmol/L) and moderate (4.6–10.0 mmol/L) are at higher risk of acute myocardial infarction [HR 1.07, (1.05–1.09) and 1.17 (1.12–1.23) respectively)]. The risk attenuated and was not significant in severe hypertriglyceridaemia (TG >10.0). All-cause mortality incrementally increased with increasing severity of hypertriglyceridaemia.

AMI, acute myocardial infarction; CVD, cardiovascular; CHD, coronary heart disease; CI, confidence interval; CVD, cardiovascular disease; HDL-C, high-density lipoprotein cholesterol; HR, hazard ratio; IHD, ischemic heart disease; LDL-C, low-density lipoprotein cholesterol; MI, myocardial infarction; OR, odds ratio; RR, relative risk; SD, standard deviation; TG, triglycerides; TG-REAL, association of hypertriglyceridaemia with all-cause mortality and atherosclerotic cardiovascular events in a Low-risk Italian population; UA: unstable angina; USA, United States of America.

^a^ A randomized, double-blind, placebo-controlled trial designed to evaluate the benefits and risks of low-dose aspirin and vitamin E in the primary prevention of cardiovascular disease and cancer in women.

Despite the strong association between hypertriglyceridaemia and ASCVD this relationship is not straightforward partly due to other lipid abnormalities, most commonly low HDL-C. Although this has been accounted for in some studies, when the confounding factor of low HDL-C is reflected, the link between hypertriglyceridaemia and ASCVD weakens (66, 69). Although ApoB is associated with proatherogenic lipoprotein particles, Apolipoprotein A1 (ApoA1) is found in anti-atherogenic particles e.g., HDL and its subfractions. The ratio of ApoB/ApoA1 have been suggested as a better predictor of ASCVD events as compared to the individual concentration of pro- and anti-atherogenic molecules (21).

#### 4.3.1 Lessons learnt from Mendelian randomisation

Epidemiological studies, while crucial for identifying associations, often face limitations in establishing causation, particularly in complex phenomena like the relationship between raised TRL and ASCVD. Confounders such as low HDL-C and others factors can obscure direct causative links. Mendelian randomization studies leveraging human genetics present a promising avenue. By exploiting genetic variants as proxies for lifelong exposure to elevated TRL, in a study of 73,513 individuals of Danish descent, genetic variants affecting levels of non fasting remnant cholesterol alone, non-fasting remnant cholesterol combined with HDL-C, HDL-C alone, and LDL-C were investigated for their impact on ischemic heart disease (IHD). The findings revealed a substantial causal risk increase for elevated nonfasting remnant cholesterol, independent of HDL-C levels, suggesting a causal association with a 2.8-fold increased risk of ASCVD. Similarly, the non fasting remnant cholesterol to HDL-C ratio showed a 2.9-fold causal risk increase. Conversely, while observational estimates indicated a 1.6-fold increased risk of ASCVD for each 1-mmol/L decrease in HDL-C, the causal estimate was inconclusive at 0.7-fold. Furthermore, for LDL-C, a 1.5-fold causal risk increase was observed, supporting its known association with ASCVD (77). These findings underscore the causal role of elevated remnant cholesterol in ASCVD development, independent of low HDL-Clevels. Similar observations were made by Do et al. (78) and others where SNPs in TG raising alleles e.g., *ANGPTL3, APOC2, APOA5, GPIHBP1, LMF1* were found to increase the risk of ASCVD, while conversely, in *APOC3* loss of function heterozygosity led to reduction in ASCVD events (79–81).

## 5 Triglycerides as residual ASCVD risk factor in statin-treated patients

Landmark statin trials that shaped our understanding of cardiovascular risk reduction with intensive LDL-C reduction still displayed residual ASCVD risk (22.4% at 2 years, 9.3% at 4.3 years, and 8.7% at 4.9 years in PROVE IT TIMI, IDEAL and TNT respectively) despite intensive statin treatment and achieving LDL-C of 1.6 mmol/L (62 mg/dl), 2.1 mmol/L (81 mg/dl) and 2.0 mmol/L (77 mg/dl) respectively (17–19). Even though efficacy of statins in primary prevention of ASCVD in type 2 diabetes mellitus (T2DM) was well demonstrated in the Collaborative Atorvastatin Diabetes Study (CARDS), 12.5% of statin recipients had an ASCVD event despite achieving median on-treatment LDL-C 2.0 mmol/L (77 mg/dl) during median follow up of 3.9 years (13). Clearly, residual ASCVD risk persists even after achieving optimal reductions in LDL-C levels in large statin trials, irrespective of the dosage. The factors that may contribute to residual cardiovascular risk are outlined in Figure 1.

A *post hoc* analysis of the PROVE IT TIMI trial focused on secondary prevention. LDL-C levels below 1.8 mmol/L (70 mg/dl) and TG levels below 2.2 mmol/L (200 mg/dl) demonstrated a 40% reduced risk of subsequent ASCVD events when compared to individuals with TG levels exceeding 2.2 mmol/L (200 mg/dl). TG <1.7 mmol/L (150 mg/dl) was independently associated with a 20% reduction in relative risk of CHD after adjustment for LDL-C and other covariates. A combination of TG <1.7 mmol/L (150 mg/dl) and LDL-C <1.8 mmol/L (70 mg/dl) was associated with the lowest ASCVD events, and each 0.1 mmol/L (10 mg/dl) lower TG concentration led to a decline in the rate of recurrent acute coronary syndrome (ACS), MI and death by 1.6% independent of LDL-C concentrations (8). Similar results were found in the intEnsive statin therapy for hypercholesteroleMic Patients with diAbetic retinopaTHY (EMPATHY) study where serum TG was associated with ASCVD regardless of the intensity of statin therapy. Each 0.1 mmol/L (10 mg/dl) increase in TG was associated with a 2.1% increased risk of ASCVD event and TG >1.5 mmol/L (135 mg/dl) was found to be an independent risk factor for developing ASCVD (82). In *post hoc* analyses of TNT and IDEAL study, non-HDL-C and ApoB were found to have a stronger association with future ASCVD events as compared to LDL-C alone. Patients with TG in the highest quantile were predicted to be at 63% increased risk as compared with the lowest quantile. This effect was attenuated but not abolished after adjustment for HDL-C. The probability of ASCVD event was 30% higher in individuals with TG levels >1.7 mmol/L (150 mg/dl), as opposed to those with levels <1.7 mmol/L (150 mg/dl). This association between elevated TG levels and an increased risk of new ASCVD events persisted in individuals with an LDL-C cholesterol concentration below 2.6 mmol/L (100 mg/dl) (9, 10). A *post hoc* analysis of dal-OUTCOMES and Myocardial Ischemia Reduction with Acute Cholesterol Lowering (MIRACL) study reported similar results, where in statin-treated patients, fasting TG was found to be independently associated with short- and long-term risk of developing ASCVD independent of LDL-C. Similar to PROVE IT-TIMI, each 0.1 mmol/L (10 mg/dl) increase in TG was associated with a 1.4%–1.6% increase in the risk of a ASCVD event (11). In all major statin trials, there is a significant residual ASCVD risk in statin recipients, which is due to atherogenic dyslipidaemia and non-LDL lipoprotein subfractions along with other contributors that constitute residual ASCVD risk profile (Figure 1).

## 6 Triglycerides and residual ASCVD risk in PCSK9 inhibitor treated patients

The FOURIER and ODYSSEY OUTCOMES trials have demonstrated significant LDL-C reduction that has translated into ASCVD risk reduction. However, despite achieving very low LDL-C, a residual risk of 9.8% and 9.5% respectively remained at 2.2 and 2.8 years respectively. Participants in both trials received moderate or high intensity statin in addition to PCSK9 monoclonal antibodies. In FOURIER, mean LDL-C at the end of the trial period was as low as 0.8 mmol/L (30 mg/dl) in the evolocumab arm. Despite this, a significant proportion of patients had ASCVD events. 9.8% of evolocumab recipients had at least one ASCVD event and 6.1% had a subsequent ASCVD event (14, 83). In a prespecified secondary analysis of the FOURIER trial, a monotonic relationship between LDL-C at 4 weeks and ASCVD events was observed, where while high LDL-C was associated with heightened risk, 10.3% of individuals achieving LDL-C <0.5 mmol/L (20 mg/dl) experienced an ASCVD event (84). TG of 1.3 mmol/L (112.3 mg/dl) at the end of the trial period along with other metabolic and inflammatory factors (Figure 1) might explain the residual ASCVD risk in this group who achieved very low LDL-C. ApoB, which constitutes a composite of all major atherogenic lipoproteins, inclusive of LDL, VLDL, IDL, remnant particles and Lp(a) would be a better therapeutic target to minimize ASCVD risk. This is supported by a recent analysis of the ODYSSEY outcome database by Hagstrom et al. where LDL-C was found to underestimate the ASCVD risk and ApoB levels were found to be a better predictor of future ASCVD events independent of LDL-C levels in a cohort of alirocumab treated patients (85). Similarly, intensive LDL-C lowering with evolocumab and statins in the “Global Assessment of Plaque Regression With a PCSK9 Antibody as Measured by Intravascular Ultrasound” (GLAGOV) study achieved a significant reduction in plaque atheroma volume (PAV) and total atheroma volume (TAV) with very low LDL-C (36.6 mg/dl, 0.9 mmol/L). Nevertheless, not all the patients achieved plaque regression. In a subgroup of participants with baseline LDL-C of 1.8 mmol/L (70 mg/dl), with a further 50%–55% reduction after treatment, 20% did not have regression in atheroma volume despite achieving very low LDL-C (86), thereby suggesting a role of TG-rich lipoproteins and other factors (Figure 1) in atheroma development and progression (87). In addition, 12.2% of evolocumab recipients had ASCVD events suggestive of residual factors other than LDL-C contributing to atherogenesis (86).

Residual ASCVD risk in statin and PCSK9-treated patients is not confined to non-LDL subfractions. Pradhan et al. have demonstrated a 62% increase in the risk of future ASCVD events (3.6% annual event rate) in statin and PCSK9-treated patients who have achieved median LDL-C of 1.07 mmol/L (41.7 mg/dl) but have raised high-sensitivity C-reactive protein (>3 mg/L) suggesting complex interplay of multiple residual ASCVD risk factors in the pathogenesis of ASCVD (88).

## 7 Therapeutic targets

The role of TG in ASCVD is well established from clinical trials (8), epidemiological (69) and Mendelian randomisation studies (24, 89). Serum TG levels are very sensitive to diet, lifestyle, and secondary factors. Three classes of drugs that preferentially reduce serum TG levels are fibrates, omega-3 fatty acids (FA) and niacin. Despite genetic studies (78) and *post hoc* analysis of landmark statin (8) and PCSK9 trials (85) suggesting a lower risk of ASCVD with reduced TG, the results from pharmacologically achieved lower TG levels with niacin, fibrates and omega-3 FA have been inconsistent. Major clinical trials of fibrates and Omega-3 FA evaluating ASCVD outcomes are summarised in Tables 2, 3 respectively.

**Table 2:** Major fibrate trials, effect on lipid profile and ASCVD outcomes.

Trial	Number of participants	Follow up (years)	Intervention	Statin	Study population	Baseline TG (mmol/L)^a^	Change in lipid profile	Outcomes	Comment
Coronary Drug Project (90)	3,892	5	Clofibrate 1.8 g OD	No	Secondary Prevention	5.8	TC: −6.5%^b^ TG: −22.3%^b^	MI/CHD Death	No difference between the composite of cardiac outcomes (28.0% vs. 30.1%) Increased risk of VTE, non-cardiovascular mortality and gallstones in the clofibrate group
Co-operative Trial (91)	7,194	5.3	Clofibrate 1.6 g OD	No	Primary Prevention	NR	NR	MI/CHD Death	20% reduction in the composite of cardiac outcomes [RR 0.80, (0.65–0.97), *p* = 0.03]. No difference in CHD death [RR 1.05, (0.66–1.68), *p* = 0.8] Increased risk of gallstones, cancer-related mortality, non-cardiovascular mortality and VTE.
HHS (92)	4,081	5	Gemfibrozil 600 mg BD	No	Primary Prevention	1.98 ± 1.33	TC: −11% LDL-C: −10% TG: −43% HDL-C: +10% Non-HDL: −14%	MI/CHD Death	Significant reduction in the composite of cardiac outcomes [RR 0.66, (0.47–0.92), *p* = 0.01] No difference in all-cause mortality.
VA HIT (93)	2,531	5.1	Gemfibrozil 600 mg BD	No	Secondary Prevention	1.82 ± 0.77	TC: −4% LDL-C: 0% TG: −31% HDL-C: +6%	MI/CHD Death	Significant reduction in composite cardiac endpoints [RR 0.80 (0.68–0.94), *p* < 0.01] No change in stroke, CV death or all-cause death.
BIP (94)	3,090	6.2	Bezafibrate 400 mg OD	No	Secondary Prevention	1.64 ± 0.58	HDL-C: +17.9% TG: −20.6% LDL-C: −6.5% TC: −4.5%	MI/CHD Death/Sudden death	No change in the composite of cardiac outcomes [RR 0.91, (0.76–1.08), *p* = 0.26] or components of the composite. Risk reduction was proportional to baseline TG and was significant only when TG >2.2 mmol/L and HDL <0.9 mmol/L (−41.8%, *p* = 0.02)
DAIS (95)	418	3	Fenofibrate 200 mg OD	No	Primary and secondary prevention in Type 2 diabetes	2.59 ± 1.39	TC: −10% LDL-C: −6% TG: −30% HDL-C: +8%	MI/PCI/CABG/Death/UA	No change in the composite of cardiac outcomes and all-cause mortality [RR 0.77, (0.53–1.13), *p* = 0.18]. Fenofibrate led to a reduced angiographic progression of coronary-artery disease in type 2 diabetes.
LEADER (96)	1,568	4.6	Bezafibrate 400 mg OD	Commencement of statin led to drop-out	Secondary Prevention	2.11 (1.53–3.01)	TC: −7.6% LDL-C: −8.1% TG: −23.3% HDL-C: +8.0%	MI/Stroke/CVD Death	No difference in composite CV outcomes [RR 0.96, (0.76–1.21), *p* = 0.72] or individual components of the composite.
FIELD (97)	9,795	5	Fenofibrate 200 mg OD	Yes	Primary and secondary prevention in Type 2 diabetes	1.95 ± 0.87	TC: −6.9% LDL-C: −5.8% TG: −21.9% HDL-C: +1.2%	MI/CHD Death	No difference in the composite of cardiac outcomes [HR 0.89, (0.75–1.05)]. Significant reduction in total cardiovascular events [HR 0.89, (0.80–0.99), *p* = 0.03] and revascularisation [HR 0.80, (0.70–0.92), *p* = 0.001] No difference in stroke [HR 0.90, (0.73–1.12), *p* = 0.3] or CV mortality (HR 1.11, 0.87–1.41, *p* = 0.4). The risk of VTE was numerically greater in fenofibrate recipients (1.6% vs. 2.4%).
ACCORD (98)	5,518	4.7	Fenofibrate 200 mg OD	Yes	Primary and secondary prevention Type 2 diabetes	1.85 (1.29 –2.62)	TC: −1.7%^b^ LDL-C: +1.4%^b^ TG: −13.5%^b^ HDL-C: +1.7%^b^	MI/Stroke /CVD Death	No change in composite CV outcome [HR 0.92, (0.79–1.08), *p* = 0.32] or components of the composite. Individuals with TG >2.33 mmol/L and HDL-C <0.88 mmol/L had numerically (12.37% vs. 17.32%) better but statistically non-significant better (*p* = 0.06) outcomes.
PROMINENT (99)	10,497	3.4	Pemafibrate 0.2 mg BD	Yes	Primary prevention with Type 2 diabetes, HDL-C <1.0 mmol/L and LDL-C <2.6 mmol/L	3.06	TC: +0.8%^b^ LDL-C: +12.3%^b^ TG: −26.2%^b^ HDL-C: +5.1%^b^	MI/Stroke/Coronary revascularisation/CVD Death	No change in composite CV outcome [HR 1.03, (0.91–1.11), *p* = 0.67] or individual components of the composite. Increase in VTE [HR 2.05, (1.35–3.17), *p* ≤ 0.001] and adverse renal events and decrease in hepatic adverse events.

ACCORD, the action to control cardiovascular risk in diabetes; BD, twice a day; BIP, bezafibrate infarction prevention study; CABG, coronary artery bypass graft; CHD, coronary heart disease; CVD, cardiovascular disease; DAIS, diabetes atherosclerosis intervention study; FIELD, fenofibrate intervention and event lowering in diabetes; HHS, Helsinki heart study; LEADER, lower extremity arterial disease event reduction; MI, myocardial infarction; OD, once daily; PCI, percutaneous coronary intervention; PROMINENT, the pemafibrate to reduce cardiovascular outcomes by reducing triglycerides in patients with diabetes; RR, relative risk; UA, unstable angina; VA-HIT, Veteran affairs high-density lipoprotein cholesterol intervention trial; VTE, venous thromboembolism.

^a^ To convert mmol/L to mg/dl – multiply by 88.57.

^b^ Between-group difference.

**Table 3:** Major omega 3 fatty acid trials, effect on lipid profile and ASCVD outcomes.

Trial	Number of participants	Follow up (years)	Intervention	EPA ± DHA (mg)	Study population	Baseline TG (mmol/L)^a^	Change in lipid profile	Outcomes	Comment
GISSI-P (100)	11,324	3.5	ω-3 FA vs. Placebo	294 + 588	Secondary Prevention	1.8	TC: +7.9% LDL-C: +9.9% TG: −3.4% HDL-C: +8.8%	MI/Stroke/CVD death	20% reduction in the composite of CV outcomes [RR 0.85, (0.68–0.95), *p* = 0.008] No difference in non-fatal CV events [RR 0.96, (0.76–1.21)
JELIS (101)	18,645	4.6	ω-3 FA + statin vs. statin alone	1,800 + 0	Primary & Secondary prevention	1.73 (1.23–2.48)	LDL-C: −25% TC: −19% TG: −9% HDL-C: +3%	MI/Revascularisation/UA/CHD Death	19% reduction in the composite of CV outcomes [HR 0.81, (0.69–0.95)]. The outcome was significant only in secondary prevention [HR 0.81, (0.66–1.00)]. No difference in primary prevention [HR 0.82, CI 0.63–1.06) or stroke [HR 1.02, (0.91–1.13)].
GISSI-HF (102)	6,975	3.9	ω-3 FA vs. Placebo	400 + 480	Symptomatic HF NYHA II-IV	1.42 (1.05–1.98)	Last TG recorded: 1.34 mmol/L (0.98–1.85)	All-cause death All-cause death/hospital admission for CVD reasons Fatal and non-fatal MI Fatal and non-fatal stroke	10–12% reduction in all-cause death [HR 0.91, (0.83–0.99), *p* = 0.04] or a composite of all-cause death and hospital admission due to CVD [HR 0.92, (0.85–0.99), *p* = 0.009] No difference in MI (HR 0.82, CI 0.63–1.06, *p* = 0.12) or stroke (HR 1.16, CI 0.89–1.51, *p* = 0.27)
Alpha Omega (103)	4,837	3.4	ω-3 FA vs. Placebo	226 + 150	Secondary prevention	1.63 (1.22–2.30)	TC: −0.30 mmol/L LDL-C: −0.37 mmol/L TG: −0.14 mmol/L HDL-C: +0.14 mmol/L	MI/Stroke/Revascularisation/CVD Death	No change in the composite of major CVD outcomes [HR 1.01, (0.87–1.17), *p* = 0.93]
OMEGA (104)	3,804	1	ω-3 FA vs. Placebo	460 + 380	Secondary prevention	NR	NR	SCD MI/Stroke/CVD Death	No change in SCD [OR 0.95, (0.56–1.60), *p* = 0.84]. No change in the composite of other CV outcomes [OR 1.21, (0.96–1.52), *p* = 0.10].
SU.FUL.OM3 (105)	2,501	4.7	ω-3 FA vs. Placebo	400 + 200	Secondary prevention	1.2 (0.9–1.6)	NR	MI/Stroke/CVD Death	No effect on major ASCVD events as composite [HR 0.90, (0.66–1.23), *p* = 0.5] or individual components of the composite.
ORIGIN (106)	12,536	6.2	ω-3 FA vs. Placebo	465 + 375	Primary and secondary prevention with Type 2 Diabetes	1.6 (1.1–2.2)	TC: −0.41 mmol/L LDL-C: −0.31 mmol/L TG: −0.26 mmol/L HDL-C: No change	CVD Death MI/Stroke/CVD Death	No effect on ASCVD death [HR 0.98, (0.87–1.10), *p* = 0.72] or composite of other CV outcomes [HR 1.01, (0.93–1.10), *p* = 0.81] or individual components of the composite.
AREDS (107)	4,203	4.8	ω-3 FA vs. Placebo	650 + 350	Primary and secondary prevention with AMD	NR	NR	MI/Stroke/CVD Death/Revascularisation/UA	No effect on major CV events as composite [HR 0.95, (0.78–1.17), *p* = 0.64] or individual components of the composite.
R&P (108)	12,513	5	ω-3 FA vs. Placebo	500 + 500	Primary and prevention with multiple CV risk factors and no history of MI	1.7 (1.2–2.2)	TC: −0.71 mmol/L LDL-C: −0.57 mmol/L TG: −0.3 mmol/L HDL-C: No change	Death from CVD/Hospitalisation for CVD	No change in CV outcomes as composite [HR 0.98 (0.88–1.08), *p* = 0.64] or components of the composite. No change in MI, Stroke or CV death as composite [HR 1.05 (0.89–1.23), *p* = 0.59] or components of the composite
ASCEND (109)	15,480	7.4	ω-3 FA vs. Placebo	460 + 380	Primary prevention in Diabetes mellitus	TG: NR Non-HDL: 2.92 ± 0.8 mmol/L	TC: −1.0%^b^ Non-HDL: −2.4%^b^ HDL-C: +1.3%^b^ TG: NR	MI/Stroke/TIA/CVD Death	No difference in CV outcomes as composite [HR 0.97, (0.87–1.08), *p* = 0.55] or individual components of composite, except vascular death [HR 0.82, (0.68–0.98)]
VITAL (110)	25,871	5.3	ω-3 FA vs. Placebo	460 + 380	Primary prevention	NR	NR	MI/Stroke/ CVD Death/revascularisation	No change in risk of CV outcomes as composite [HR 0.93, (0.82–1.04)] regardless of concomitant use of other LLT or diabetes. 17% reduction in MI/CHD [HR 0.83, (0.71–0.97)].
REDUCE-IT (111)	8,179	4.9	ω-3 FA vs. Placebo (olive oil)	4,000 + 0	Primary (with diabetes) & Secondary prevention in TG 1.5–5.6 mmol/L and LDL-C 1.0–2.6 mmol/L	2.4 (2.0–3.1)	TC: NR LDL-C: −6.6%^b^ TG: −14.1%^b^ HDL-C: −3.0%^b^	MI/Stroke/UA/Revascularisation/CVD Death	Significant reduction in composite CV outcomes [HR 0.75, (0.68–0.83), *p* < 0.001] and individual components of the composite. NNT to prevent 1 event was 21 over 4.9 years. In the subgroup, the risk reduction was significant if TG >1.7 mmol/L and for secondary prevention.
STRENGTH (112)	13,078	3.5	ω-3 FA vs. Placebo (Corn oil)	1,860 + 1,500	Primary (with diabetes) or secondary prevention, LDL-C <2.6 mmol/L, TG 2.0–5.6 mmol/L	2.7 (2.2–3.5)	TC: −3%^b^ LDL-C: +3%^b^ TG: −18%^b^ HDL-C: +1%^b^	MI/Stroke/UA/Revascularisation/CVD Death	No change in the composite of CV outcomes [HR 0.99, (0.90–1.09), *p* = 0.84] or individual components of composite endpoints. The null effect persisted in subgroup analysis based on diabetes, TG levels and primary or secondary prevention

AMD, age related macular degeneration; AREDS, the age-related eye disease study; ASCEND, a study of cardiovascular events in diabetes; CHD, coronary heart disease; CI, confidence interval; CV, cardiovascular; CVD, cardiovascular disease; DHA, docosahexaenoic acid; EPA, eicosapentaenoic acid; FA, fatty acids; GISSI-HF, Gruppo Italiano per lo Studio della sopravvivenza nell’infarto miocardico – heart failure; GISSI-P, gruppo Italiano per lo studio della sopravvivenza nell’infarto miocardico- prevenzione trial; HDL-C, high density lipoprotein cholesterol; HR, hazard ratio; JELIS, Japan EPA lipid intervention study; LDL-C, low density lipoprotein cholesterol; MI, myocardial infarction; NNT, number needed to treat; NYHA, New York Heart Association; OR, odds ratio; ORIGIN, outcome reduction with an initial glargine intervention; PUFA, polyunsaturated fatty acids; R&P, risk and prevention study; REDUCE-IT, reduction of cardiovascular events with icosapent ethyl–intervention trial; RR, relative risk; SCD, sudden cardiac death; STRENGTH, long-term outcomes study to assess statin residual risk with epanova in high cardiovascular risk patients with hypertriglyceridemia; SU.FUL.OM3, supplémentation en folates et omega-3; TC, total cholesterol; TG, triglycerides; TIA, transient ischemic attack; UA, unstable angina; VITAL, the vitamin D and omega-3 trial.

^a^ To convert mmol/L to mg/dl – multiply by 88.57.

^b^ Between-group difference.

### 7.1 Diet and lifestyle

TG levels are highly responsive to dietary interventions and physical activity; therefore, the first line of intervention is often diet and lifestyle modifications (38). Epidemiological and clinical trial data substantiate the correlation between the Mediterranean-style dietary pattern and reduction in TG levels (113, 114). Modification of macronutrient composition through dietary interventions, adopting low-carbohydrate diets, and implementing caloric restriction have shown efficacy in improving TG (115). Notably, the Mediterranean diet emerges as the dietary pattern with the most consistent and robust evidence supporting its efficacy in addressing hypertriglyceridaemia (113). Additionally, among dietary components, the consumption of omega-3 FA has been the subject of a substantial number of RCTs that have consistently demonstrated their effectiveness in reducing TG levels (116, 117). Sea food and oily fish are a rich source of polyunsaturated fatty acids (PUFA) that not only lead to a significant decrease in TG but also improve blood pressure, systemic inflammation and increase HDL-C (118). In the Framingham Heart Study Offspring Cohort, individuals in the highest quintile for the Mediterranean-style diet exhibited the lowest TG levels over a 7-year follow-up (119). Additionally, these interventions offer ancillary benefits such as weight loss and reduced waist circumference. A comprehensive approach to lifestyle modification, encompassing dietary strategies with an emphasis on reduced carbohydrate and saturated fat intake, regular physical activity, and weight management, can result in substantial TG reductions ranging from 20% to 50%.

### 7.2 Niacin

While there exists evidence substantiating the correlation between elevated TG and a heightened susceptibility to ASCVD events, a multitude of clinical trials assessing pharmacological interventions aimed at reducing TG levels have not demonstrated reduction in ASCVD events. The Atherothrombosis Intervention in Metabolic Syndrome with Low HDL-C/High Triglycerides: Impact on Global Health Outcomes (AIM-HIGH) study investigated the impact of niacin on individuals on intensive statin therapy with elevated TG levels and low HDL-C levels. Despite niacin achieving a 31% reduction in TG and 21% increase in HDL-C in a cohort with baseline LDL-C <1.8 mmol/L (70 mg/dl), no difference in the composite primary cardiovascular endpoint emerged between the niacin-administered cohort and the control group (120). Similar results were replicated later by the Heart Protection Study 2–Treatment of HDL to Reduce the Incidence of Vascular Events (HPS2-THRIVE), which investigated the combined administration of niacin and laropiprant, a prostaglandin D2 receptor 1 antagonist, with simvastatin and/or ezetimibe (121). A systematic review and metanalysis spanning the preceding six decades and 17 RCTs failed to show ASCVD risk reduction with niacin treatment across all patient cohorts, including those on statin treatment (122). Consequently, niacin has been withdrawn from the European market.

### 7.3 Fibrates

Fibrates are synthetic ligands for peroxisome proliferator-activated receptor (PPAR) alpha receptors, through which they exert lipid-lowering and pleiotropic effects via increasing lipolysis by upregulating LPL activity, hepatic FA uptake, increased production of ApoA1, inhibition of apolipoprotein C3 and reduced expression of various pro-inflammatory cytokines and adhesion molecules (123). Whilst hypertriglyceridaemia is deemed as a risk factor for ASCVD and current guidelines recommend employing additional measures to reduce TG <1.7 mmol/L (150 mg/dl) to mitigate cardiovascular risk (75, 124), the addition of fibrates to statins to achieve this has not shown any additional benefit (Table 2). The history of employing fibrates to reduce cardiovascular risk dates to the 1950s when a group of farm workers were found to have low cholesterol after being exposed to an insecticide (phenyl ethyl acetic acid) that led to the synthesis of its analogue, clofibrate (125, 126). When clofibrate was employed for the first time in a primary prevention cohort, although showing a significant reduction in nonfatal MI, it failed to demonstrate a significant benefit in cardiovascular mortality. Furthermore, it increased the risk of gallstones and non-cardiovascular mortality (91) which precluded its use in the modern era. Whilst the outcomes from clofibrate were disappointing, primary, and secondary prevention trials towards the end of 20th century with another fibrate, gemfibrozil demonstrated significant benefits in reducing cardiovascular events (92, 127). The secondary prevention trial (VA HIT) included patients only with HDL-C <1.0 mmol/L (39 mg/dl) thereby signalling the benefit of fibrates in individuals with atherogenic dyslipidaemia (high TG, low HDL-C). Intriguingly, the ASCVD benefit was attributed to increased HDL-C rather than a reduction in TG levels. Subgroup analysis of the Helsinki Heart Study (HHS) trial echoed similar results where the greatest benefit was derived in individuals with low HDL-C (<1.08 mmol/L, 42 mg/dl) and high TG (>2.3 mmol/L, 204 mg/dl) (128). The predominant effect of fibrates in ameliorating ASCVD risk in atherogenic dyslipidaemia, characterized by high TG and low HDL-C, has been replicated in subsequent trials with bezafibrate and fenofibrate where, although these drugs failed to demonstrate significant ASCVD risk reduction across the whole cohort, participants with atherogenic dyslipidaemia derived maximum benefit (94, 98) (Table 2). Several meta-analyses have demonstrated ASCVD risk reduction with fibrates only in the setting of atherogenic dyslipidaemia (129, 130). In addition to ASCVD risk reduction, fibrates reduce the progression of diabetic retinopathy (131, 132).

More recently, a selective PPAR alpha receptor modulator, pemafibrate, has been evaluated for ASCVD risk reduction in hypertriglyceridaemia in a subset of patients with LDL-C <1.8 mmol/L (70 mg/dl) whilst on statins or <2.6 mmol/L (100 mg/dl) in cases of statin intolerance. In addition, the study was focused on individuals with atherogenic dyslipidaemia i.e., with T2DM, TG >2.2 mmol/L (195 mg/dl) and HDL-C <1.0 mmol/L (38 mg/dl). Two-thirds of the study population had prior ASCVD events. This cohort was representative of modern-day residual ASCVD risk profile where intensive LDL-C reduction and background statin therapy have been employed. Pemafibrate is distinct from other fibrates as it is a selective PPAR receptor modulator. Though the lipid-modifying effect of pemafibrate is comparable with fenofibrate, pemafibrate has superior pleiotropic effects and increases HDL's cholesterol efflux capacity *in vitro* (133). Nevertheless, despite better pharmacokinetics and achieving a significant reduction in TG, VLDL-C and remnant particles and a comparable HDL-C increase compared to earlier fibrate trials, no significant reduction in major ASCVD events was demonstrated, thereby casting doubt on the utility of TG reduction via fibrates on ASCVD events in statin-treated patients with adequately lowered LDL-C. Safety analysis of pemafibrate also revealed that drug recipients were twice as likely to suffer deep vein thrombosis (DVT) and pulmonary embolism (PE) compared to placebo. Similar findings have been reported with fenofibrate [FIELD trial, RR for venous thromboembolism (VTE) 1.5 (1.1–2.0)] (97) and clofibrate [Coronary Drug Project, RR for PE 1.8 (1.1–2.8)] (90). The association of fibrates with VTE has been supported by a French pharmacovigilance database and other case-control studies (134–137). The reason for the increased risk of VTE is not known, though an increased level of homocysteine with or without other contributing factors might explain the increased risk (137).

Kim and colleagues have recently conducted a meta-analysis and meta-regression analysis of 12 RCTs, including Pemafibrate to Reduce Cardiovascular Outcomes by Reducing Triglycerides in Patients with Diabetes (PROMINENT) trial, employing fibrates for ASCVD risk reduction. While authors demonstrated overall reduction in ASCVD risk, mainly in secondary prevention group, it was found that reduction in ASCVD events was significantly associated with reduction in LDL-C. Each 1 mmol/L reduction in LDL-C level was associated with a reduction in major adverse cardiovascular events (MACE) with a relative risk (RR) 0.71 (95% CI 0.49–0.94, *p* = 0.01). On the other hand, a reduction in TG concentration was not associated with a significant reduction in MACE. Change in LDL-C was found to be the main driver of heterogeneity between the studies (138). Fibrates can reduce LDL-C, however this LDL-C lowering potential is inconsistent (139) and is significantly dampened with concomitant use of statins (140). In the meta-analysis by Kim et al. both baseline and LDL-C change were inversely correlated with the year of publication, suggesting better lipid control post-statin era and hence diminished efficacy of fibrates in reducing LDL-C (138). There also appears a negative correlation between change in LDL-C and baseline LDL-C with Pemafibrate (141). sdLDL are more atherogenic than large buoyant LDL particles. Using Sampson formula to calculate sdLDL-C, no difference has been shown in its concentration between pemafibrate and placebo group (142). Likely, low baseline LDL-C levels diminish the role of TG in sdLDL formation. Additionally, Pemafibrate stimulates hepatic TG lipase, potentially enhancing sdLDL production, which could offset the decrease in sdLDL attributed to lower TG levels. In the PROMENENT study Pemafibrate reduced remnant cholesterol and TG, however increased levels of ApoB, a surrogate indicator for LDL particle count. Given the strong correlation between ApoB levels and sdLDL-C levels, the lack of reduction in estimated sdLDL-C levels in the PROMINET trial is not unexpected (142). The beneficial impact of Pemafibrate on ASCVD might be restricted to individuals with hypertriglyceridemia those with relatively higher LDL-C and would be interesting to investigate the effect of Pemafibrate in a sub-cohort of PROMINENT trial who had higher baseline LDL-C. Moreover, recent advancements in the treatment of diabetes and hypertension, along with the growing utilization of cardioprotective anti-hyperglycaemic medications, high-intensity statins, along with addition of other potent LDL-C lowering drugs may have reduced the remaining cardiovascular risk to such an extent that it becomes challenging to discern notable differences in outcomes solely through triglyceride lowering strategies.

In summary, the use of fibrates to mitigate ASCVD risk was supported by early trials with the greatest benefit being derived for patients with atherogenic dyslipidaemia. However, results from the PROMINENT study (99) along with an increased propensity to develop VTE with fibrates have cast doubt over their clinical utility for ASCVD risk reduction and they should be administered cautiously in patients, particularly in individuals at high risk of VTE.

### 7.4 Omega 3 fatty acids

Omega 3 FA are PUFA that cannot be synthesised by humans. They are found in abundance in seafood and hence are also called marine fatty acids. Our understanding of the relationship between increased consumption of PUFA, favourable lipid profile and reduce incidence of CHD dates to the 1970s when Greenland Inuit whose diet was rich in seafood were found to have favourable metabolic profiles as compared to Danish controls (143). Subsequently, several potential mechanisms via which omega-3 FA can reduce the burden of ASCVD independent of its lipid-lowering potential have been proposed (144). Despite this, clinical studies have produced divergent results for ASCVD outcomes with omega-3 FA supplementation (Table 3).

The initial landmark trial of omega-3 FA, Gruppo Italiano per lo Studio della Sopravvivenza nell'Infarto miocardico- Prevenzione trial (GISSI-P) ignited excitement when it showed a 15%–20% reduction in fatal and non-fatal ASCVD events (100). Similar results were reproduced in Japan EPA lipid Intervention Study (JELIS) where Eicosapentaenoic acid (EPA) supplementation led to a 19% reduction in major ASCVD events (101). Nonetheless, this earlier excitement waned when subsequent trials failed to reproduce ASCVD benefits (Table 3). Positive outcomes in GISSI-P and JELIS could be due to the diet and lifestyle of the study population from Italy and Japan respectively who consume seafood on a more regular basis and hence may have higher circulating omega-3 FA levels. Attained level of blood EPA is an important factor and may explain the positive outcome of JELIS trial. This perception is supported by findings from two recent trials, Reduction of Cardiovascular Events with Icosapent Ethyl–Intervention Trial (REDUCE-IT) (111) and Long-Term Outcomes Study to Assess Statin Residual Risk with Epanova in High Cardiovascular Risk Patients with Hypertriglyceridemia (STRENGTH) (112), where outcomes for REDUCE-IT, like JELIS, were positive after using a higher dose of purified icosapent ethyl (IPA) (4 g/day) leading to a greater increment in its blood levels. However, outcomes were neutral for STRENGTH, where the increment in EPA levels was lower as compared to REDUCE-IT. Baseline EPA level in study participants in JELIS was 97 µg/ml which was significantly higher than REDUCE-IT and STRENGTH, (26.1 µg/ml and 21.0 µg/ml respectively) but achieved EPA levels after supplementation with omega-3 FA were comparable between JELIS (1.8 g/day of purified IPA, 70% increase, 169 µg/ml) and REDUCE-IT (4 g/day of purified IPA, 394% increase, 144 µg/ml) which produced positive outcomes. In STRENGTH however, the achieved EPA levels after supplementation [EPA 1,860 mg + docosahexaenoic acid (DHA) 1,500 mg/day] led to a 269% increment yet the absolute achieved EPA levels remained lower (89.6 µg/ml) than the baseline EPA levels of JELIS participants (101, 111, 112). This suggests employing higher doses of EPA may help in achieving an “effective therapeutic level” of circulating EPA to reduce ASCVD events. Moreover, the mean baseline TG level in JELIS participants was 1.7 mmol/L (150 mg/dl) which, according to current guidelines is defined as normal (38). This, along with ASCVD risk reduction disproportionate to the amount of TG reduction and failure of fibrates to reduce ASCVD events despite achieving greater TG reduction suggests independent pathways through which EPA exerts its antiatherogenic effects. Trials demonstrating neutral outcomes employed a combination of EPA and DHA while trials demonstrating positive ASCVD outcomes employed purified EPA. This might suggest that any beneficial effect conferred by EPA is partially neutralised by DHA, though there is no mechanistic data to suggest any proatherosclerotic and/or prothrombotic effects of DHA. One plausible explanation could be the low absolute dose of EPA used in EPA + DHA as compared to the higher one used in EPA monotherapy (without DHA) studies.

EPA and DHA are two distinct molecules that have diverse effects on membrane integrity, stability, and cholesterol distribution (145). While EPA preserves membrane structure, DHA increases membrane fluidity and has fewer antioxidant properties that wane more quickly as compared to EPA (146). The antioxidant properties of EPA exceed quantitatively those of fibrates which might explain positive ASCVD outcomes despite proportionately less TG reduction (146). The differential interaction of EPA and DHA with the cell membrane, cholesterol distribution, formation of cholesterol crystals and atherosclerotic plaque, antioxidant capacity and modulation of endothelial dysfunction (147) might explain the ASCVD protection conferred by EPA-based therapeutics. The application of the INSPIRE biobank registry (formerly known as the Intermountain Heart Collaborative Study) afforded a unique opportunity to explore the relationship between spontaneously acquired levels of omega-3 metabolites and the occurrence of long-term major adverse cardiovascular events (MACE) within a diverse cohort of high-risk individuals encompassing both primary and secondary prevention populations referred for angiography (148). The findings substantiated the observed cardioprotective impact linked to elevated circulating and acquired levels of EPA, as opposed to DHA. Notably, these results suggested that increased DHA levels and a resultant reduced EPA/DHA ratio might diminish the cardiovascular protective effect of EPA. Elevated plasma concentrations of EPA and the combined EPA + DHA demonstrated a protective effect against incident MACE. However, unadjusted DHA alone did not display a correlation with incident MACE or a protective effect. Furthermore, DHA, when adjusted for EPA, exhibited an almost twofold increased risk of MACE for individuals in the highest quartile compared to the lowest quartile of DHA. These findings, in conjunction with reduced attained EPA serum levels, may contribute to understanding the outcomes observed in recent trials, such as the STRENGTH and REDUCE-IT (148). The Randomized Trial for Evaluation in Secondary Prevention Efficacy of Combination Therapy - Statin and Eicosapentaenoic Acid (RESPECT-EPA) was a recent open label trial focussed on patients with secondary prevention on background statin therapy. After 6 years, a marginally significant reduction in the primary cardiovascular outcome (10.9% vs. 14.9%, hazard ratio 0.785, *p* = 0.0547) and a significant decrease in the composite secondary endpoint (8.0% vs. 11.3%, hazard ratio 0.734, *p* = 0.0306) was observed. The trial employed same dose of EPA as in JELIS but the baseline EPA level was half of that of JELIS participants (45 µg/ml vs. 97 µg/ml) and focus on patients with higher chronic inflammation suggested by lower EPA:AA (arachidonic acid) ratio (149, 150). Though the details of the study are awaited, like JELIS, higher baseline EPA levels in a Japanese cohort with subsequent higher clinically meaningful levels after treatment might suggest that absolute serum EPA levels govern ASCVD outcomes whereas patients with low baseline EPA might require higher doses of EPA to achieve clinically significant levels. Similar observations had been made in a sub-study of REDUCE-IT where achieved EPA levels in the treatment group were found to be associated with ASCVD events, heart failure and cardiovascular death (151).

### 7.5 Weight loss and bariatric surgery

The common dyslipidaemia associated with obesity is marked by elevated TG levels and low HDL-C. Most diet and lifestyle interventions accompanied by some degree of weight loss are translated into improved hypertriglyceridaemia (152). Pharmacological interventions with glucagon like peptide 1 (GLP1) and gastric inhibitory polypeptide (GIP) receptor agonists are associated with a 20%–25% reduction in TG accompanied by 15%–20% weight loss (153, 154). Bariatric surgery (BS) offers another option to attain significant and sustained weight loss that not only improves hypertriglyceridaemia but also improves the qualitative composition of lipoprotein particles (155). Incidence of hypertriglyceridaemia was significantly reduced after bariatric surgery during the follow up period of 2 years (156) where TG level remains the strongest univariate predictor of mortality in the Swedish Obese Subject (SOS) study (157). In a metanalysis of 178 studies, recipients of BS demonstrated a significant decrease in mean TG levels as compared to both baseline and non-surgical controls. Reduction in TG varied depending on type of BS employed with the greatest reduction observed with Roux-en-Y gastric bypass (RYGB), but each procedure displayed significant reductions compared to baseline and controls (158). We have demonstrated significant reductions in TG along with other atherogenic lipoproteins, markers of systemic inflammation and insulin resistance after BS in patients with and without diabetes (159–162). In T2DM the susceptibility to ASCVD is significantly increased by the existence of microvascular disease that may manifest as nephropathy, neuropathy, or retinopathy (163). Hypertriglyceridaemia is associated with small nerve fibre damage and cardiac autonomic neuropathy (117, 155, 164, 165). Our findings, along with those of others, indicate evidence of small nerve fibre regeneration post-bariatric surgery. Additionally, we established a correlation between improvements in neuropathic parameters and reductions in TG levels (160, 166, 167). Notably, the beneficial effects on small nerve fibre structure and function extend beyond patients with T2DM. A similar association of hypertriglyceridaemia is noted in relation to retinopathy and nephropathy (38).

### 7.6 Others

In addition to lipid-modifying therapy targeting serum TG, several other therapeutic agents have been or are in the process of development, targeting various potential mediators of residual cardiovascular risk with variable success (Table 4). Careful selection of patients, after addressing traditional modifiable risk factors based on clinical features, laboratory values, risk of adverse effects, co-morbidities and patient preferences can aid in defining the choice of novel therapy. The absolute benefit gained by these add-on novel therapies largely depends upon the baseline residual risk after addressing conventional modifiable risks.

**Table 4:** Therapeutic targets for reducing atherosclerotic cardiovascular disease (ASCVD) risk: summary of clinical trials and outcomes.

Therapeutic target	Drug(s)	Trial	Outcome	Comment
Inflammation	Darapladib	STABILITY (168)	Primary Outcome: Nonfatal myocardial infarction, nonfatal stroke, or cardiovascular death HR: 0.94 (0.85–1.03); *p* = 0.20	A significantly greater number of patients on the drug experienced side effects that led to discontinuation.
	Canakinumab	CANTOS (169)	Primary Outcome: Nonfatal myocardial infarction, nonfatal stroke, or cardiovascular death HR: 0.85 (0.74–0.98); *p* = 0.02	hsCRP and IL6 were reduced by 52.4% and 30.9% with baseline hsCRP 4.2 mg/dl. no effect on lipid profile. Increased risk of neutropenia and thrombocytopenia. Reduced risk of arthritis, gout, and cancer-related mortality.
	Methotrexate	CIRT (170)	Primary outcome: Nonfatal myocardial infarction, nonfatal stroke, or cardiovascular death HR: 1.01 (0.82−1.25); *p* = 0.91	No change in hsCRP, IL1β or IL-6. Baseline hsCRP was low (1.5 mg/L)
	Colchicine	COLCOT (171)	Primary Outcome: MI, coronary revascularisation, stroke, cardiac arrest, CVD Death HR: 0.77 (0.61 −0.96), *p* = 0.02	10.3% reduction in hsCRP from a baseline of 4.28 mg/L. Pneumonia and GI side effects were more in the treatment group.
		LoDoCo2 (172)	Primary Outcome: CV death, MI, stroke, coronary revascularisation HR: 0.69 (0.57–0.83); *p* < 0.001	Laboratory indicators of inflammation were not reported. No difference in infection, pneumonia, neutropenia, or GI side effects were reported between groups
Thrombosis	Rivaroxaban	ATLAS ACS 2-TMI 51 (172)	Primary Outcome: Nonfatal myocardial infarction, nonfatal stroke, or cardiovascular death HR: 0.84 (0.74–0.96), *p* = 0.008	31% reduced risk of stent thrombosis. Rivaroxaban increased the risk of ICH, and major and minor bleeding unrelated to revascularisation.
		COMPASS (174)	Primary Outcome: Nonfatal myocardial infarction, nonfatal stroke, or cardiovascular death HR (Rivaroxaban + Aspirin vs. Aspirin): 0.76 (0.66−0.86) *p* < 0.001 HR (Rivaroxaban vs. Aspirin): 0.90 (0.79−1.03) *p* = 0.12	Whilst no difference in fatal bleeding or ICH between groups, the risk of major bleeding was more in rivaroxaban recipients in both the comparison cohorts. The net clinical benefit was lower in Aspirin + Rivaroxaban as compared to aspirin but comparable between aspirin and rivaroxaban monotherapy.
	Dual antiplatelets	PEGASUS-TIMI 54 (175) (After 1 year of MI)	Primary Outcome: Nonfatal myocardial infarction, nonfatal stroke, or cardiovascular death HR (Aspirin + Ticagrelor 90 mg BD vs. Aspirin: 0.85 (0.75−0.96); *P* = 0.008 HR (Aspirin + Ticagrelor 60 mg BD vs. Aspirin): 0.84 (0.74–0.95) *p* = 0.004	Whilst no difference in fatal bleeding or ICH between groups, the risk of major and minor bleeding was more in ticagrelor recipients.
		THEMIS (176) (no prior history of MI or stroke)	Primary Outcome: Nonfatal myocardial infarction, nonfatal stroke, or cardiovascular death HR: 0.9 (0.81–0.99), *p* = 0.04	The risk of traumatic ICH and major bleeding was greater in ticagrelor, with no difference in spontaneous ICH between groups.
Lp(a)	Pelacarsen	HORIZON (177)	Active phase 3 study to establish the reduction of cardiovascular risk in patients with established ASCVD and elevated Lp(a)	
	Lipoprotein Apheresis	MultiSELECT (178)	Active prospective observational study to evaluate the clinical benefit of Lp(a) reduction using lipoprotein apheresis on ASCVD.	
	LY3473329	KRAKEN (179)	Recruiting for Phase II trial to evaluate the efficacy and safety of LY3473329 in adults with elevated Lp(a) at high risk for cardiovascular events.	
	Olpasiran	OCEAN(a) (180)	Active phase 3 study to compare the effect olpasiran to placebo, on the risk for CHD death, MI, or urgent coronary revascularization in participants with ASCVD and elevated Lp(a)	
HDL-C	Anacetrapib	REVEAL (181)	Primary Outcome: MI, CHD or Coronary revascularisation RR: 0.91 (0.85–0.97) *p* = 0.004	Anacetrapib was the only CETP inhibitor that demonstrated positive ASCVD outcomes. However, the development of anacetrapib was halted due to concerns about the prolonged accumulation of the drug in adipose tissue.
	Niacin	AIM-HIGH (120)	Primary Outcome: CHD Death, MI, Ischemic Stroke, Hospitalisation for ACS, or revascularisation HR: 1.02 (0.87–1.21)	Niacin recipients had a net 15.2% increase in HDL and a 20.5% reduction in TG and no side effects as compared to placebo.
	CSL112	AEGIS II (182)	Active Phase III trial to evaluate the efficacy and safety of CSL112 in reducing the risk of MACE in patients with ACS.	
	Obecetrapib	PREVAIL (183)	Active phase 3 study to establish the reduction of MACE in patients with established ASCVD maximum LLT and LDL-C >1.8 mmol/L (70 mg/dl) and TG <4.5 mmol/L (400 mg/dl)	

ACS, acute coronary syndrome; AEGIS II, study to investigate CSL112 in subjects with acute coronary syndrome; AIM HIGH, the atherothrombosis intervention in metabolic syndrome with low HDL-C/high triglycerides: impact on global health outcomes; ASCVD, atherosclerotic cardiovascular disease; ATLAS ACS 2-TMI 51, Anti-Xa therapy to lower cardiovascular events in addition to standard therapy in subjects with acute coronary syndrome–thrombolysis in myocardial infarction 51; CANTOS, canakinumab anti-inflammatory thrombosis outcome study; CETP, cholesteryl ester transfer protein; CHD, coronary heart disease; CIRT, cardiovascular inflammation reduction trial; COLCOT, colchicine cardiovascular outcomes trial; COMPASS, cardiovascular outcomes for people using anticoagulation strategies; HORIZON, assessing impact of lipoprotein (a) lowering with Pelacarsen on major cardiovascular events in patients with CVD; HR, hazard ratio; hsCRP, high sensitivity CRP; ICH, intracranial haemorrhage; IL, interleukin; KRAKEN, efficacy and safety of oral once-daily LY3473329 in adults with elevated lipoprotein(a) at high risk for cardiovascular events; Lp(a), lipoprotein (a); MACE, major adverse cardiovascular events; MI, myocardial infarction; MultiSELECt, effect of lipoprotein (a) elimination by lipoprotein apheresis on cardiovascular outcomes; PEGASUS TIMI 54, prevention of cardiovascular events in patients with prior heart attack using ticagrelor compared to placebo on a background of aspirin–thrombolysis in myocardial infarction 54; REVEAL, randomized evaluation of the effect of anacetrapib through lipid modification; STABILITY, stabilization of atherosclerotic plaque by initiation of darapladib therapy; THEMIS, the effect of ticagrelor on health outcomes in diabetes mellitus patients intervention study.

## 8 Conclusion

Historically ASCVD risk reduction measures have predominantly been LDL-centric. Despite significant strides in developing LDL-C lowering agents and their proven benefits in reducing ASCVD risk, a substantial portion of the secondary prevention cohort remains undertreated. Factors such as clinical inertia, discrepancies in access to effective lipid-lowering therapies, and challenges in implementing guidelines contribute to this problem. Even with optimal guideline-based treatment, lipid-related but also lipid-independent residual risk remains a significant contributor to recurrent events, emphasizing the need to identify atherogenic targets beyond LDL-C.

TRL as a risk factor for ASCVD have gained much attention recently supported by epidemiological, genetic, and mechanistic studies. Addressing this TRL-associated risk is challenging, given mixed results from clinical outcome studies evaluating various therapeutic approaches. Fibrates had previously been shown to be of benefit in atherogenic dyslipidaemia but recent results from the PROMINENT trial have cast doubt on their utility in ASCVD risk reduction. Further, increased risk of VTE has been reported inconsistently in earlier fibrate trials and therefore merits careful consideration. ASCVD risk reduction from REDUCE-IT and RESPECT EPA but not from STRENGTH and other studies employing combined EPA and DHA suggest TG-independent pathways to mitigate ASCVD risk with purified EPA products. Patients at high risk of recurrent ASCVD events may benefit from employing additional therapeutic agents to target components of the residual cardiovascular risk profile.
